# Plausibility of Using a Checklist With YouTube to Facilitate the Discovery of Acute Low Back Pain Self-Management Content: Exploratory Study

**DOI:** 10.2196/23366

**Published:** 2020-11-20

**Authors:** Andrey Zheluk, Jess Maddock

**Affiliations:** 1 School of Biomedical Sciences Charles Sturt University Bathurst Australia; 2 University of Sydney Menzies Centre for Health Policy Sydney Australia; 3 iCCnet SA SA Health Adelaide Australia

**Keywords:** YouTube, low back pain, lower back pain, self-management, social media, infodemiology, infodemic, quality of health information

## Abstract

**Background:**

Access to guideline-consistent effective care for acute low back pain (ALBP) is generally regarded as limited. Researchers have recognized the potential of YouTube as a clinical and patient education resource that may improve access to appropriate care. However, the heterogeneity of evaluation approaches and variable quality of health information have generally limited the potential of YouTube as a self-management intervention.

**Objective:**

This study aims to increase the understanding of ALBP content available on YouTube in 2020 and to establish the plausibility of using a simple checklist to facilitate the discovery of YouTube content consistent with current guidelines. We examined the following 4 research questions: how was the data set defined, what are the metadata characteristics of the videos in the data set, what is the information quality of ALBP YouTube videos, and what are the characteristics of the YouTube data set based on an ALBP self-management checklist?

**Methods:**

This was an exploratory, qualitative infodemiology study. We identified videos in our data set through YouTube search based on popular ALBP-relevant search terms identified through Google Trends for YouTube. We accessed YouTube metadata using the YouTube data tools developed by the University of Amsterdam. We used a modified Brief DISCERN checklist to examine the information quality. We developed a checklist based on the 2018 Lancet Low Back Pain guidelines to examine self-management content.

**Results:**

We analyzed a data set of 202 YouTube videos authored by chiropractors, physicians, physiotherapists, and instructors of yoga and other disciplines. We identified clear differences in the ALBP videos in our data set based on the authors’ disciplines. We found that the videos authored by each discipline strongly featured a specific intervention domain, that is, education, treatment, or exercise. We also found that videos authored by physicians were consistently coded with the highest ALBP self-management content scores than all other disciplines.

**Conclusions:**

The results returned by YouTube in response to a search for back pain–related content were highly variable. We suggest that a simple checklist may facilitate the discovery of guideline-concordant ALBP self-management content on YouTube. Further research may identify the clinical contexts in which the use of an ALBP checklist with YouTube is feasible.

## Introduction

### Background

This exploratory study aims to increase the understanding of the acute low back pain (ALBP) content available on YouTube (Google Inc) in 2020 and to establish the plausibility of using a simple checklist to facilitate the discovery of YouTube content consistent with current guidelines. Low back pain exerts a high economic and social burden across the globe. The 2018 Global Burden of Disease study suggested that low back pain was the leading cause of years lived with disability in most countries [1]. 

This paper focuses on ALBP. ALBP is commonly described as a new onset or exacerbation incident lasting less than 12 weeks and including sciatica [2,3]. Most people with ALBP have rapid improvements in pain and disability within several weeks. In most cases, the cause of low back pain cannot be identified, and most low back pain is therefore described as nonspecific [4,5]. However, pain and disability persist for a proportion of people. Up to 80% of people with ALBP may experience recurrence within 12 months [6,7]. The estimates of recovery from an episode of low back pain over 12 months range between 54% and 90% [8,9]. Differing definitions of recovery also complicate the epidemiology of ALBP. Stanton et al [10] described the heterogeneity of case definitions of acute exacerbations of preexisting back pain as further contributing to the diverse understanding of the scale and outcomes of ALBP.

### Access to Guideline-Consistent ALBP Care

Access to guideline-consistent effective care for ALBP is generally regarded as limited in the scientific literature. We have described access to ALBP care based on a framework developed by Aday and Anderson [11]. Aday and Anderson’s 4 dimensions of access are characteristics of the health delivery system, utilization of health services, characteristics of the population at risk, and consumer satisfaction. These are described below.

#### Characteristics of the Health Delivery System and Utilization of Services

In 2018, the Lancet Low Back Pain Series Working Group suggested that usual care for back pain was generally unnecessary [12]. The authors defined usual care as incorporating complex pain medications, spinal imaging, spinal injections, hospitalization, and surgical procedures. Among the factors identified as contributing to health system–related access distortions are financial incentives for low value care; clinician attitudes [13,14]; and poor adherence to guidelines by emergency departments [15], family physicians [14,16], and allied health providers [17,18].

#### Patient Characteristics

The social determinants of health are associated with reduced access to health services [19]. Researchers have also described lower socioeconomic status as a predisposing factor for low back pain [20].

#### Patient Satisfaction

Researchers have suggested that imaging, extensive testing, and other nonguideline-based investigations and interventions are largely driven by patient demand [21]. Systematic reviews suggest that ALBP patient satisfaction is generally associated with physical examination, diagnosis and prognosis, exclusion of serious pathology, pain relief, and functional improvements [22]. Furthermore, failure by clinicians to provide expected nonguideline-based care may reduce satisfaction and adherence to clinician-prescribed self-management recommendations [23].

In summary, access to effective ALBP care is a complex challenge for patients, clinicians, and policy makers.

### Self-Management and Self-Care

Most people manage low back pain with little assistance from health care providers. Estimates of the number of individuals that manage back pain independently, or with occasional formal health care, range from 50% to 70% [24,25]. In scientific literature, the terms *self-management* and *self-care* are often used interchangeably. Self-management is generally regarded as a clinician-guided collaborative intervention that enhances an individual’s capacity to monitor and manage their own physical and emotional responses and maintain their quality of life [26,27]. The related term self-care generally refers to actions and decisions taken independent of health providers [28]. Importantly, self-management and self-care are not passive processes. Rather, these processes involve active patient decision making, including symptom monitoring, goal setting, information search and interpretation, and self-efficacy [29,30]. In the case of ALBP, psychosocial status, including fear avoidance, self-efficacy [31,32], and catastrophizing [33], may contribute to the transition from an acute to a chronic condition. Therefore, access to psychosocial support is a consideration in ALBP self-management interventions.

### Digital Health Interventions

Digital health interventions (DHIs) may improve access to guideline-consistent ALBP care by facilitating self-management decision making. DHIs include all interventions delivered via digital technologies that facilitate health behavior change [34]. This includes web search, social media, symptom checkers, apps, and telehealth. The reported access benefits of DHIs include reduced health system costs, patient waiting, travel time, and expenses [35] and improved patient-provider communication [36], health outcomes [37], scalability, and safety [38].

By 2020, searching the internet was regarded as a routine dimension of individual health self-management. However, access to web-based health information remained more limited among older and low-income people [39]. Researchers have noted further potential problems associated with the feasibility of implementing DHIs at scale. These feasibility problems include acceptability and demand, usability, real-world implementation, and integration with existing practices [40]. In the case of DHIs for ALBP, a 2017 systematic review found no evidence of positive clinical outcomes or cost-effectiveness [41].

Researchers have suggested that increased patient access to health information represents a profound change in the relationship between patients and providers [42]. Digitally enabled independent self-care by patients has not been universally welcomed by health providers [43,44]. Researchers have reported a consistent pattern of health care provider unease when managing patients informed by internet information.

### Importance of YouTube

YouTube is one of the world’s most popular websites. In 2020, YouTube was the second most visited website [45] and the second most popular social media network globally [46]. YouTube is commonly used as a source of instructional advice. In 2018, Pew Research reported that 86% of adults in the United States used YouTube for “figuring out how to do things they haven’t done before” [47]. It is the instructional use of YouTube that is relevant to this study.

### Approaches Used to Analyze YouTube Clinical Videos

YouTube has been widely researched as a source of health information. Although researchers have generally described the potential of YouTube as a clinical and patient education resource, they have also noted the heterogeneity of evaluation approaches and variable quality of clinical information. We identified 3 broad research approaches for evaluating YouTube health videos. These approaches are (1) metadata characteristics, (2) information quality review, and (3) expert clinician review. In practice, most YouTube research has incorporated two or more of these approaches.

First, researchers have described the metadata characteristics of YouTube videos exclusive of the content. A systematic review by Sampson et al [48] described that the most common video characteristics included in studies were the number of views, video length, likes, date posted, and language of the video. Similarly, a 2018 systematic review found number of views, video duration, and likes and dislikes to be the top 3 characteristics reported in evaluations of YouTube [49]. Other researchers [50] have suggested that view counts were the second most frequently cited concept in assessing quality on YouTube.

Second, reviews have reviewed the information quality of videos using validated instruments. These instruments are generic health information quality assessment tools, and they commonly describe the credibility of sources and information contained within publications. Commonly used instruments for evaluating the quality of health information incorporated in YouTube videos include DISCERN [51], Brief DISCERN [52], Patient Education Materials Assessment Tool [53], Health on the Net Foundation Code of Conduct [54], Flesch-Kincaid reading level [55], and guidelines from the *Journal of the American Medical Association* [56].

A third approach involves the evaluation of video content by expert clinicians. Expert clinicians have generally described the potential of YouTube as a self-management resource in positive terms [57]. Similarly, clinician reviewers have consistently noted concerns regarding the discovery of accurate clinical content. Clinician reviewers noted specific concerns about the selection of appropriate search terms [58], including the influences of algorithms [59], and video popularity (views) [60,61] as particular challenges in discovering accurate content.

In summary, the evaluation of YouTube videos is an underdeveloped area. Although there are several approaches to evaluating YouTube content, these are all time consuming, relatively complex, and thus unsuitable for shared care discussions in a clinical setting. Similarly, many patients turn to the internet for self-management advice, although they lack simple cues to identify the content that is consistent with current clinical guidelines.

## Methods

### Overview

This study was conducted to increase the understanding of YouTube information about ALBP in advance of potential feasibility and clinical studies. This exploratory study aims to (1) increase the understanding of the ALBP content available on YouTube in 2020 and (2) establish the plausibility of using a simple checklist to facilitate the discovery of YouTube content consistent with current ALBP guidelines. We defined plausibility as “a scenario is one that fits prior knowledge well, with many different sources of corroboration, without complexity of explanation, and with minimal conjecture” [62].

We examined the following 4 research questions: how was the data set defined?, what are the metadata characteristics of the videos in the data set?, what is the information quality of ALBP YouTube videos?, and what are the characteristics of the YouTube data set based on an ALBP self-management checklist?

This study incorporates 3 approaches to evaluate YouTube health content. These approaches are (1) analysis of metadata characteristics, (2) analysis of information quality, and (3) expert clinician review. In this study, we extended these approaches by classifying YouTube content based on author’s professional discipline and substituted a simple checklist for clinician expertise to analyze ALBP self-management content.

We used 2 simple checklists to analyze the YouTube content within a defined data set. Checklists have been widely used in medicine to reduce costs and improve health outcomes [63]. Coding of all items in the data set was conducted by the 2 authors of this study. Intercoder reliability for all coding results was reviewed using Krippendorf alpha [64] (Multimedia Appendix 1).

This is an infodemiology study. Eysenbach described infodemiology as “the science of distribution and determinants of information” [65]. Infodemiology studies have primarily examined public health and policy issues [66]. In contrast, this study examined patterns of YouTube clinical information, with the ultimate aim of developing a novel clinical intervention for the self-management of ALBP.

### RQ1: How Was the Data Set Defined?

#### Step 1: Selection of Appropriate Search Terms

Search is the most common approach for finding content on YouTube [67]. We identified the search terms “back pain” and “lower back pain” as popular relevant YouTube search terms using Google Trends for YouTube [68] in Australia and the United States over the 5-year period 2015 to 2020. The third term “back pain exercises” was selected for both popularity and self-management relevance (Multimedia Appendix 2). These search terms were aimed at identifying ALBP content likely to be viewed by YouTube audiences in March 2020. However, no raw search volumes are available in Google Trends for YouTube. This means that a direct comparison of search volumes at the population scale is not possible. Furthermore, back pain epidemiology is imprecise, and the term “back exercises” is not relevant to ALBP epidemiology. Therefore, we used another high-burden disease, diabetes, as a search volume comparator in YouTube. Searches for diabetes and back pain on YouTube were comparable in Australia and the United States for the period between 2015 and 2020.

#### Step 2: Characteristics of Raw Data Set

We downloaded the metadata for the 300 top-rated English language videos across the 6 search categories in our raw data set in March 2020. The search term categories are described in Table 1. We used the video list module from the YouTube Data Tools (YTDT) developed by the University of Amsterdam to extract these metadata as individual comma separated values (CSV) format files for each of the 6 search categories [69]. The YTDT extract data directly from the YouTube application programming interface and make them available for download as a CSV file. The fields contained in the metadata include the number of views of each video at the specified date, length, internet address, publisher, and date of publication.

We aimed to account for YouTube Search personalization and algorithmic selection in our data set. By default, YouTube displays search results based on relevance. However, YouTube algorithms also modify search results with reference to personalized search history [70] and most viewed videos [71]. To account for relevance and popularity, we separately downloaded the YTDT metadata for the top 50 *most relevant* videos for each of the 3 search terms “back pain,” “lower back pain,” and “back exercises.” We repeated this for the top 50 *most viewed* videos for each of the 3 search terms. Our raw data set thus consisted of 300 English language videos. These videos were divided across 6 separate search term categories, with 50 videos in each category. (See Table 1 and Multimedia Appendix 3).

**Table 1:** Percentage of videos from each discipline by search term, views, and relevance in raw data set.

Discipline	Lower back pain relevance, n (%)	Lower back views, n (%)	Back pain relevance, n (%)	Back pain views, n (%)	Back exercises relevance, n (%)	Back exercises views, n (%)
Chiropractic	6 (12)	7 (14)	6 (12)	16 (32)	5 (10)	2 (4)
Fitness	3 (6)	8 (16)	1 (2)	7 (14)	26 (52)	30 (60)
Medicine	11 (22)	4 (8)	12 (24)	3 (6)	1 (2)	1 (2)
Physiotherapy	21 (42)	11 (22)	25 (50)	7 (14)	18 (36)	13 (26)
Yoga	0 (0)	9 (18)	0 (0)	10 (20)	0 (0)	2 (4)
Other	9 (18)	11 (22)	6 (12)	7 (14)	0 (0)	2 (4)

#### Step 3: Validation of the Raw Data Set

By default, YTDT returns the US search results. The authors of this study were located in Australia. We were uncertain about how YouTube geographic and personalization algorithms influenced YTDT or YouTube search results in the location of the study. We therefore validated the YTDT results against the results from YouTube from Sydney, Australia, and from New York, United States. To do this, we first removed the personalization and geographic identifiers from our YouTube website search results. To remove personalization and geographic identifiers, we used a Chromebook with factory reset, Chrome browser with no sign in, and virtual private network (VPN) to link first to New York and second to Sydney. We separately recorded the results for the top 50 *most relevant* filtered videos for each of the 3 search terms “back pain,” “lower back pain,” and “back exercises.” We repeated this for the top 50 *most viewed* videos for each of the 3 search terms. We then compared the YTDT raw data results with the New York and Sydney YouTube website results.

#### Step 4: Cleansing the Raw Data Set to Produce the Final Data Set

We identified multiple identical videos repeated across the 300 videos across the 6 separate categories in the YTDT raw data set. After removing the duplicates, we retained 202 unique videos across the 6 search categories. These 202 unique videos were pooled to form the final data set.

### RQ2: What are the Metadata Characteristics of the Videos in the Final Data Set?

We examined the metadata characteristics of each video in the final data set. First, we coded the 202 unique videos in the final data set according to the author’s stated disciplinary affiliation. We used the following 6 disciplinary categories: chiropractor, fitness, medical doctor, physiotherapist, yoga, and other (including osteopaths and massage therapists; Table 1 and Multimedia Appendix 4). Researchers have identified relationships between author’s disciplines and user assessments of source credibility. The assessment of web-based source credibility is generally based on rapid evaluation of multiple content features, including visual design [72], trustworthiness and expertise of the source [73], and social cues such as likes and comments [74]. Source credibility is a dimension of user engagement with video content and thus relevant to this study. Second, we coded each video according to the 3 content categories: education, real-time exercise, or real-time treatment. These categories were derived from the videos in the data set. Third, we incorporated YTDT data, including length of video, number of views, and YouTube channel name. Through this approach, we were able to describe the characteristics of the final data set by the author’s professional discipline.

### RQ3. What is the Information Quality of ALBP YouTube Videos?

We used a modified Brief DISCERN instrument to assess the information quality of the final data set for this study. The full DISCERN instrument has been widely used in YouTube research [75]. The 6-question Brief DISCERN was designed to be a simpler version of the full DISCERN for patient and clinician use [76]. The Brief DISCERN has been used to evaluate the quality of web-based health content [77]. However, we were not able to identify previous peer-reviewed research using the Brief DISCERN for YouTube analysis.

To analyze information quality, we first modified the Brief DISCERN instrument. We added an ALBP self-management–specific codebook to the original 6 items in the Brief DISCERN instrument (Multimedia Appendix 5). Second, we coded each video in the final data set using our modified Brief DISCERN instrument. Each video was coded as *yes* or *no* only. Third, we organized results by authors’ disciplines. Thus, we used the unvalidated modified Brief DISCERN to analyze the information quality of videos in the final data set by the authors’ discipline.

### RQ4: What are the Characteristics of the YouTube Data Set Based on an ALBP Self-Management Checklist?

We analyzed the final data set based on the checklist of ALBP self-management strategies that we developed for this study. We included checklist items that an individual patient may reasonably be expected to independently implement as part of a self-management intervention for ALBP. In contrast to generic information quality YouTube evaluation tools such as DISCERN, this checklist incorporates specific ALBP self-management guideline-consistent items.

First, we developed a codebook for analyzing the data set using the ALBP checklist. The ALBP checklist was based on self-management items described in the Lancet 2018 ALBP guidelines [12] (Multimedia Appendix 6). This includes maintaining physical activity, education, identification of red flags, analgesia, and reassurance. Second, we coded each video in the data set using the ALBP self-management checklist. Third, we analyzed the ALBP checklist results by authors’ disciplines. In summary, by examining the characteristics, information quality, and self-management content, we aimed to determine whether it was plausible that a checklist for YouTube video assessment may facilitate self-management of ALBP.

## Results

### Overview

We identified clear differences based on the author’s discipline in the ALBP videos in our data set. We found that the videos authored by each discipline strongly featured a specific intervention domain such as education, treatment, or exercise. Using a checklist, we found that the videos authored by physicians were consistently coded with the highest ALBP self-management content scores relative to all other disciplines. We suggest that a checklist may facilitate the discovery of guideline-consistent ALBP YouTube content.

### RQ1: How Was the Data Set Defined?

We compared the YTDT results with the Australian and US website results to determine the validity of the YTDT raw data set. We found that the US raw data set obtained via YTDT matched with the Australian and US YouTube website results obtained via anonymous sign in and VPN through Chromebook from New York and Sydney. After the removal of duplicates, we identified 202 unique videos. These 202 videos became our final data set (Multimedia Appendix 8). The final data set represented popular videos likely to be displayed to YouTube searchers for information on back pain in Australia and the United States in March 2020.

### RQ2: What are the Metadata Characteristics of the Videos in the Data Set?

We had several notable findings from our analysis of the characteristics of the final data set. Videos published by mainstream health providers (physicians and physiotherapists) were more common in results filtered by *search relevance* than in *most viewed* categories (Table 1 and Multimedia Appendix 4). However, overall, ALBP videos published by other providers (chiropractors, fitness, yoga, and other categories) were viewed more often than mainstream health provider videos (Textbox 1 and Multimedia Appendix 7). Overall, chiropractic videos were the most viewed discipline in our final data set. We found that each discipline predominantly produced videos in a specific domain. For example, medical authors primarily published education videos, whereas chiropractors published primarily real-time treatment videos (Table 2 and Multimedia Appendix 8). The most viewed video in our data set featured real-time chiropractic treatment (Multimedia Appendix 9). This video scored poorly on the modified Brief DISCERN and ALBP checklists. User comments suggested that this video was commonly viewed for the purposes of sexual gratification. In summary, we identified clear differences in the ALBP videos in our data set based on the author’s discipline. The disciplinary background of the ALBP video author appears to be a noteworthy consideration in selecting guideline-consistent YouTube videos appropriate for facilitating the self-management of ALBP.

**Video views by discipline (mean [SD]).**

Chiropractic

- 5,946,902 (sample SD 11,566,170)

Fitness

- 2,161,920 (sample SD 1,638,935)

Medicine

- 2,731,637 (sample SD 3,770,147)

Physiotherapy

- 1,526,882 (sample SD 2,527,643

Yoga

- 4,822,096 (sample SD 2,868,535)

Other

- 6,465,767 (sample SD 9,298,051)

**Table 2:** Intervention domain by discipline (final data set).

Intervention domain	Number of videos	Education, n (%)	Exercise, n (%)	Treatment, n (%)
Chiropractic	27	2 (7)	6 (22)	19 (70)
Fitness	54	1 (2)	53 (98)	0 (0)
Medicine	20	20 (100)	0 (0)	0 (0)
Physiotherapy	66	5 (8)	60 (91)	1 (2)
Yoga	11	0 (0)	11 (100)	0 (0)
Other	24	1 (4)	5 (22)	18 (78)

### RQ3. What is the Information Quality of ALBP YouTube Videos?

We used a modified Brief DISCERN checklist to examine the information quality of ALBP videos in the data set. We examined the information quality for each discipline separately (ie chiropractic, fitness, medicine, physiotherapy, yoga, and *other* categories). The number of videos in the final data set varied by discipline. We therefore displayed information quality results by each modified Brief DISCERN item as a percentage of the number of *yes* responses to that item (Table 3 and Multimedia Appendix 10). For example, in the medicine discipline, 75% (n=15) of videos were coded yes in response to Question 3 of the modified Brief DISCERN. Question 3 refers to videos featuring a biologically plausible mainstream explanation of the mechanism of treatment. In the case of the *medicine* category, 75% (n=15) of the videos were coded *yes* for providing a biologically plausible mainstream explanation of the mechanism of treatment action.

**Table 3:** Results of modified Brief DISCERN coding.

Intervention domain	Information sources, n (%)	When was the information published, n (%)	How it works? n (%)	Benefits, n (%)	Risks, n (%)	Overall quality of life, n (%)
Chiropractic	0 (0)	0 (0)	2 (6)	29 (94)	1 (3)	7 (23)
Fitness	1 (2)	11 (2)	0 (0)	15 (28)	1 (2)	8 (15)
Medicine	1 (5)	2 (10)	15 (75)	18 (90)	6 (30)	16 (80)
Physiotherapy	3 (5)	2 (3)	9 (14)	44 (67)	3 (5)	17 (26)
Yoga	0 (0)	0 (0)	0 (0)	11 (100)	0 (0)	11 (100)
Other	1 (4)	0 (0)	3 (13)	12 (52)	2 (8)	7 (30)

Overall, we found that videos categorized as *medicine* were consistently coded with higher scores than all other disciplines. These higher scores indicated that medically authored videos had the highest information quality. In contrast, videos from *fitness* and *other* disciplines were consistently coded with the lowest information quality scores.

### RQ4: What are the Characteristics of the YouTube Data Set Based on an ALBP Self-Management Checklist?

We used the ALBP self-management checklist to examine the content for each discipline in the final data set. Overall, we found that medically authored videos were coded with consistently higher scores for self-management content than all other disciplines (Table 4 and Multimedia Appendix 11). Chiropractic and fitness videos were consistently coded with the lowest scores for ALBP self-management. Overall, we found that the ALBP self-management checklist may be more sensitive than the modified Brief DISCERN instrument in assisting researchers in identifying differences in self-management content among disciplines and among individual videos.

In summary, we identified clear differences in the ALBP videos in our data set based on the author’s discipline. The author’s discipline appeared to be a determinant of the number of views, information quality, and ALBP self-management content of the videos in the data set.

**Table 4:** Results of acute low back pain checklist coding.

Intervention domains	Acute, n (%)	Activities of daily living, n (%)	Analgesia, n (%)	Red flag, n (%)	Affect, n (%)	Appropriate prognosis, n (%)
Chiropractic	9 (29)	10 (32)	9 (29)	4 (13)	13 (42)	5 (16)
Fitness	18 (33)	4 (7)	1 (2)	9 (17)	19 (35)	4 (7)
Medicine	19 (95)	16 (80)	15 (75)	13 (65)	19 (95)	19 (95)
Physiotherapy	52 (79)	18 (27)	19 (29)	32 (48)	42 (64)	19 (29)
Yoga	11 (100)	5 (45)	8 (73)	0.00	11 (100)	11 (100)
Other	14 (61)	5 (22)	7 (30)	3 (13)	20 (87)	5 (22)

## Discussion

### Principal Findings

We identified considerable variability in the guideline concordance of ALBP self-management content on YouTube. We found that the video author’s discipline is an indicator of the provision of guideline-consistent information. We suggest that the access to guideline-consistent ALBP content may be improved by referring to the author’s discipline. Furthermore, we suggest that a checklist used with YouTube videos may facilitate the discovery of guideline-consistent ALBP self-management content. We have described the implications of our findings under the following categories: access and discovery, discipline-specific discovery, and self-management.

#### Access and Discovery

YouTube is a widely available and popular channel for health information. YouTube is free, multilingual, and easy to navigate, without commercial or professional gatekeepers. It is a visual medium demanding low literacy [78]. YouTube is a popular source of instructional advice [47]. Therefore, researchers have described the positive potential of YouTube as a patient resource [57]. YouTube has the potential to improve access to guideline-consistent self-management advice, consistent with patient preferences.

This study suggests that, in practice, the discovery of guideline-consistent ALBP self-management content on YouTube is a health access challenge. YouTube is not primarily a source of self-management health advice. It is a commercial platform directed at increasing viewing time [79]. To increase viewing time, YouTube constantly recommends different videos based on an individual’s prior search history and personalized search algorithms.

Researchers and media have suggested that YouTube algorithms promote misinformation, including health misinformation [80,81]. During 2020, widespread concerns about social media dissemination of misinformation on COVID-19 led to YouTube both actively monitoring and restricting pandemic-related content [82]. However, the exceptional information environment present during the pandemic is unlikely to be replicated for all health conditions or for ALBP.

Health researchers have proposed several approaches for improving patient access to guideline-consistent YouTube self-management content. These approaches include encouraging health organizations and clinicians to increase their engagement with YouTube content [83], the use of celebrities in videos [61], shared clinical decision making based on YouTube content [84], algorithmic interventions [81,85], and direct government intervention [86]. On the basis of this study, condition-specific checklists may offer a potential approach to improve access to guideline-consistent ALBP self-management content.

#### Discipline-Specific Discovery

The YouTube video author’s discipline may have implications for health access and content discovery. We identified consistent differences in information quality and ALBP self-management content between disciplines represented in our data set (Table 4). We believe that this may have implications for the discovery and use of ALBP content. The patient’s perceptions of author’s discipline may be reinforced by web-based source credibility effects [83,87]. For example, medical videos about ALBP may be regarded as more authoritative than chiropractic videos. The YouTube author’s discipline may thus cue patients to specific ALBP self-management content. Although we did not assess the effects of source credibility, we believe that this dimension of YouTube health content warrants further investigation.

Author’s discipline is a predictor of the content of an individual video. We found that 100% (N=20) of the medical videos were coded as primarily educational content, 70% of the chiropractic videos were real-time treatments, and 90% of physiotherapist-authored videos consisted primarily of exercise content (Table 2). This suggests that author’s discipline may also be used to guide clinicians when considering the selection of guideline-consistent educational and exercise content for ALBP self-management interventions.

#### Self-Management and Shared Care

The variability of YouTube health content is generally described as having negative implications for the self-management and self-care of health conditions. Within peer-reviewed health literature, inaccurate content and poor-quality information sources are often described as misleading to YouTube viewers. However, self-management and self-care are not passive patient processes [29,30]. Researchers have suggested that these processes involve active patient decision making, incorporating active information search and interpretation, symptom monitoring, goal setting, and self-efficacy.

Engagement with YouTube videos should not be considered a passive or uncritical process. In the case of health content specifically, viewers may be motivated to exert additional cognitive effort during decision making [88,89]. The additional individual cognitive effort may be characterized as the *Ikea effect* [90]. The Ikea effect suggests that individuals’ task engagement and self-efficacy may be enhanced through personalization and discovery. Similar guided discovery effects have been noted in educational research [91]. In the case of ALBP, clinician guidance of video discovery based on patient preferences may facilitate effective self-management.

Simple checklists may improve clinician engagement with internet-informed patients. Clinician engagement with internet-informed patients is frequently described as poor [43,44]. Clinician engagement with individual patients over video content may enhance adherence to self-management recommendations. The reported benefits of clinician-guided use of YouTube for self-management include reinforcement of emotional support and clinician advice [92], preclinical screening [93,94], and as a substitute for clinician advice in instances of poor communication during clinical interactions [36]. An ALBP-specific checklist may cue patients to specific domains in advance of health care encounters and provide the foundation for more positive shared care engagements focused around ALBP internet content. Under these conditions, clinicians may choose to actively recommend web-based content rather than defensively respond to patient questions.

In summary, we suggest that the discovery of an appropriate YouTube video for ALBP self-management is highly variable. This is consistent with the existing literature on YouTube health content. However, the use of an ALBP checklist by clinicians may plausibly facilitate access to guideline-consistent ALBP self-management content. For clinicians, an ALBP checklist may also facilitate engagement with internet-informed patients.

### Limitations

We identified several limitations in this study.

#### Limitations of YouTube Geolocation Data

This research was conducted in Australia. We used US YouTube data to investigate English-language YouTube viewing behavior based on global viewing statistics. Country-specific YouTube views data for individual videos are not available in the public domain. Similarly, Google Trends YouTube data are not directly comparable across countries as it is normalized and displayed as percentages only [95]. These YouTube limitations restrict the potential matching with epidemiological, health insurance, waiting times, policy, and other population data sources. However, studies within a specific geographic location that focus on the use of YouTube for self-management of health conditions could incorporate these dimensions into study designs and results.

#### Limited Examination of Metadata

The YTDT metadata tool contains multiple metadata fields describing each video, including all available comments. In this study, we used only the URL, channel name, and video length. We believe video recommendations within YouTube merit investigation for evaluating video popularity and engagement, consistent with research conducted by Zhou et al [96]. Analysis of YouTube comments has been identified as a rich source of data. Analysis of comments and other metadata was beyond the scope of this study.

#### Scope of YouTube Content

The analysis in this paper was based exclusively on existing publicly available YouTube content. In addition to YouTube, there are multiple commercial ALBP apps and video content available on proprietary distribution channels. The analysis of health provider commercial and proprietary content was outside the scope of this study.

#### Digital Divide as an Access Consideration

We did not examine differential access to YouTube based on age, income, or ethnicity. Future research examining the feasibility of YouTube in clinical settings should examine access to YouTube by older and low-income people.

### Further Research

Further research is needed to establish the feasibility of checklists and YouTube content discovery for self-management of ALBP in specific clinical contexts. We propose 2 directions for future research to extend this exploratory research into clinical practice. First, a future research program could refine the questions of feasibility, including cost-effectiveness and clinical utility. We suggest that the next stage of research on the use of YouTube for self-management of ALBP could focus on establishing feasibility in a specific clinical context, such as low acuity low back pain interventions by paramedics. Second, during 2020, COVID-19 accelerated the demand and supply for telehealth across the globe [97]. This has also accelerated the experimentation with novel clinical approaches. In light of the rapid uptake of telehealth during 2020, further research into the feasibility of incorporating YouTube with telehealth self-management may be warranted.

### Conclusions

Individuals are increasingly using YouTube to self-diagnose and self-manage health conditions, including ALBP. However, the results returned by YouTube in response to searches for back pain content were highly variable. This exploratory study aims to increase the understanding of the ALBP content available on YouTube in 2020 and to establish the plausibility of using a simple checklist to facilitate the discovery of YouTube content that is consistent with current management guidelines. We suggest that a simple checklist may facilitate the discovery of guideline-consistent ALBP self-management content on YouTube. Further research may identify the clinical contexts in which the use of an ALBP checklist with YouTube is feasible.

## Appendix

Multimedia Appendix 1 Krippendorf alpha intercoder reliability.

Multimedia Appendix 2 Google Trends YouTube. Relevant terms.

Multimedia Appendix 3 Percentage of videos from each discipline by search term, views, and relevance in raw data set.

Multimedia Appendix 4 Video views by discipline (mean).Final data set.

Multimedia Appendix 5 Intervention domain by discipline. Final data set.

Multimedia Appendix 6 Modified Brief Discern code book.

Multimedia Appendix 7 ALBP checklist code book.

Multimedia Appendix 8 Final data set.

Multimedia Appendix 9 Most viewed video in final data set.

Multimedia Appendix 10 Results of modified Brief Discern coding.

Multimedia Appendix 11 Results of ALBP checklist coding.

## Abbreviations

ALBP: acute low back pain
CSV: comma separated values
DHI: digital health intervention
RQ: research question
VPN: virtual private network
YTDT: YouTube data tools
