# Supplementary material for: Plausibility of Using a Checklist With YouTube to Facilitate the Discovery of Acute Low Back Pain Self-Management Content: Exploratory Study
Source: JMIR Form Res. 2020 Nov 20;4(11):e23366. doi: 10.2196/23366 (PMC7718094; doi:10.2196/23366)
Supplement: Multimedia Appendix 1 [file formative_v4i11e23366_app1.pdf]

Appendix 4: Krippendorf's alpha intercoder reliability

Items reviewed. N=104 out of 201 items in final data set.

Modified Brief Discern - Krippendorf Alpha					
	Alpha	Coder 1	Disagreement	Coder 2	Total items
1. Is it clear what sources of information were used to compile the publication?	1	104	0	104	208
5. Is it clear when the information used or reported in the publication was produced?	1	104	0	104	208
9. Does it describe how each treatment works?	1	104	0	104	208
10. Does the publication describe the benefits of each treatment?	1	104	0	104	208
11. Does it describe the risks of each treatment?	1	104	0	104	208
13. Does it describe how the treatment choices affect overall quality of life?	1	104	0	104	208

Items reviewed. N=104 out of 201 items in final data set.

ALBP Checklist - Krippendorf Alpha					
	Alpha	Coder 1	Disagreement	Coder 2	Total items
1. Acute	0.930746	101	3	104	208
2. ADL	0.957721	102	2	104	208
3. Analgesia	0.935252	101	3	104	208
4. Red flag	0.935252	101	3	104	208
5. Affect	0.97734	103	1	104	208
6. Prognosis	1	104	0	104	208
