# Supplementary material for: Plausibility of Using a Checklist With YouTube to Facilitate the Discovery of Acute Low Back Pain Self-Management Content: Exploratory Study
Source: JMIR Form Res. 2020 Nov 20;4(11):e23366. doi: 10.2196/23366 (PMC7718094; doi:10.2196/23366)
Supplement: Multimedia Appendix 2 [file formative_v4i11e23366_app2.pdf]

Appendix 5: Google Trends Youtube. Relevant terms.

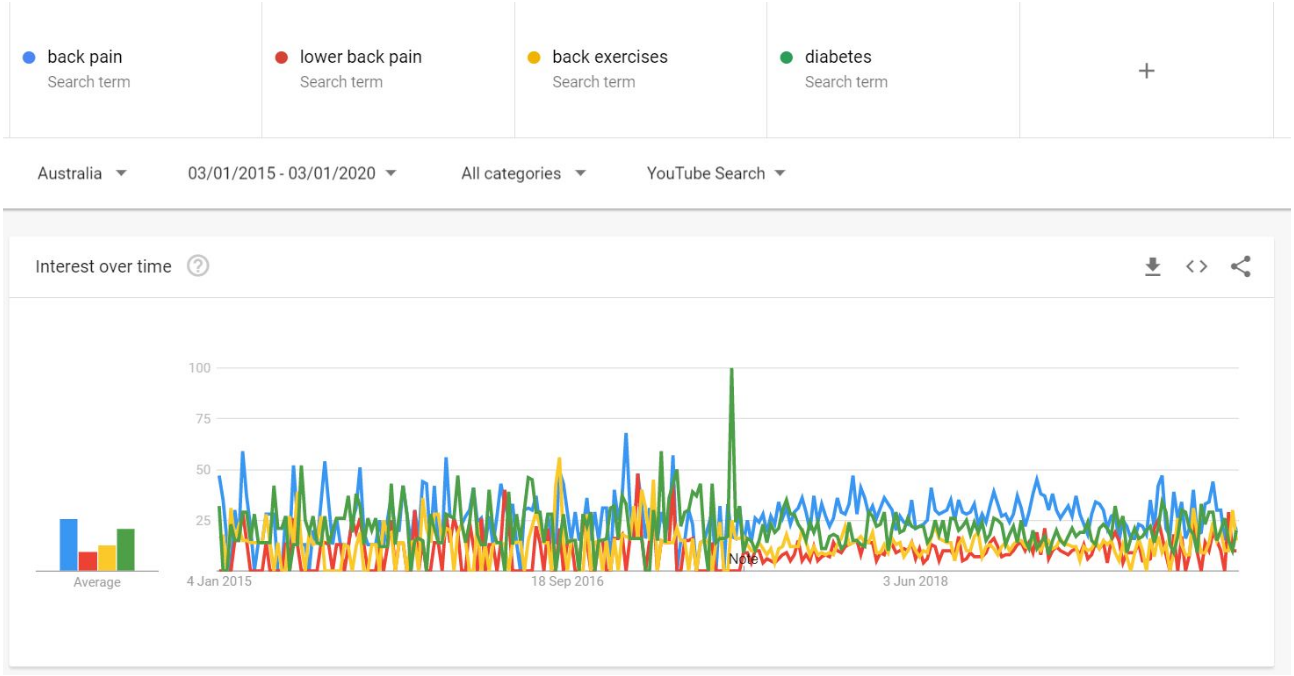

Google Trends YouTube search 2015-2020. United States.

Search terms: back pain, lower back pain, back exercises . Diabetes as comparator

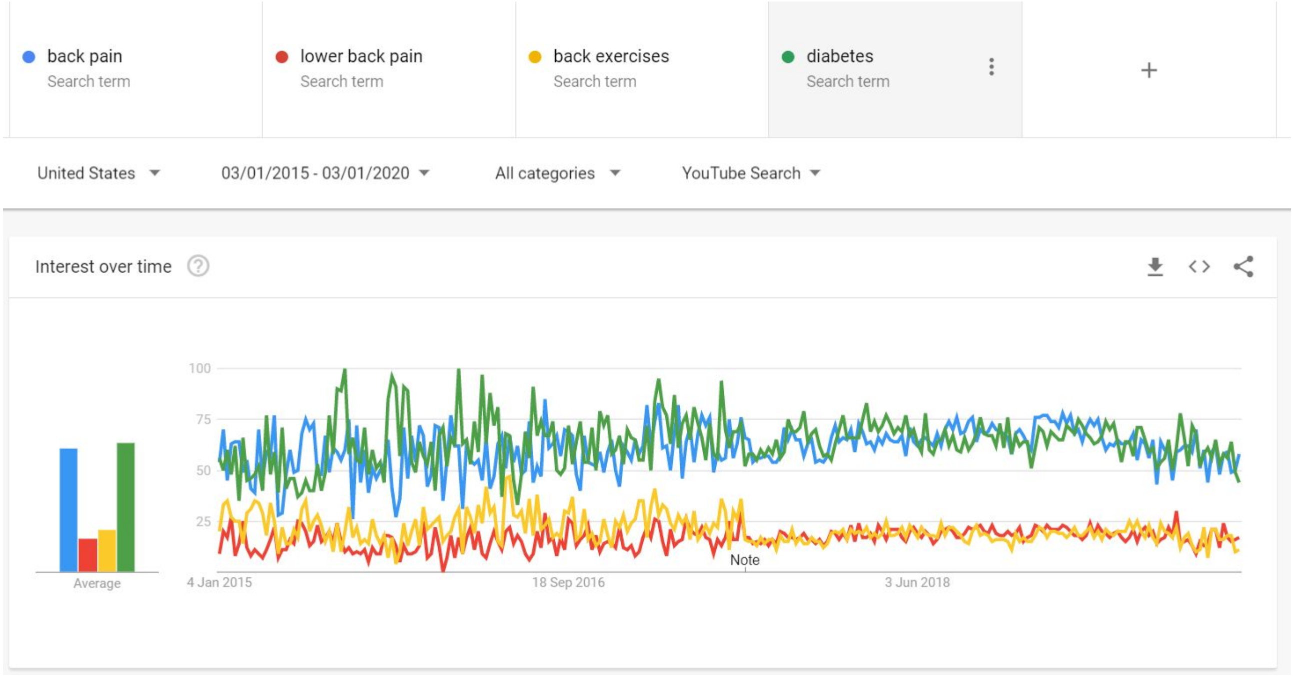

Google Trends YouTube search 2015-2020. Australia.

Search terms: back pain, lower back pain, back exercises . Diabetes as comparator
