# Supplementary figures and images for: Plausibility of Using a Checklist With YouTube to Facilitate the Discovery of Acute Low Back Pain Self-Management Content: Exploratory Study
Source: JMIR Form Res. 2020 Nov 20;4(11):e23366. doi: 10.2196/23366 (PMC7718094; doi:10.2196/23366)

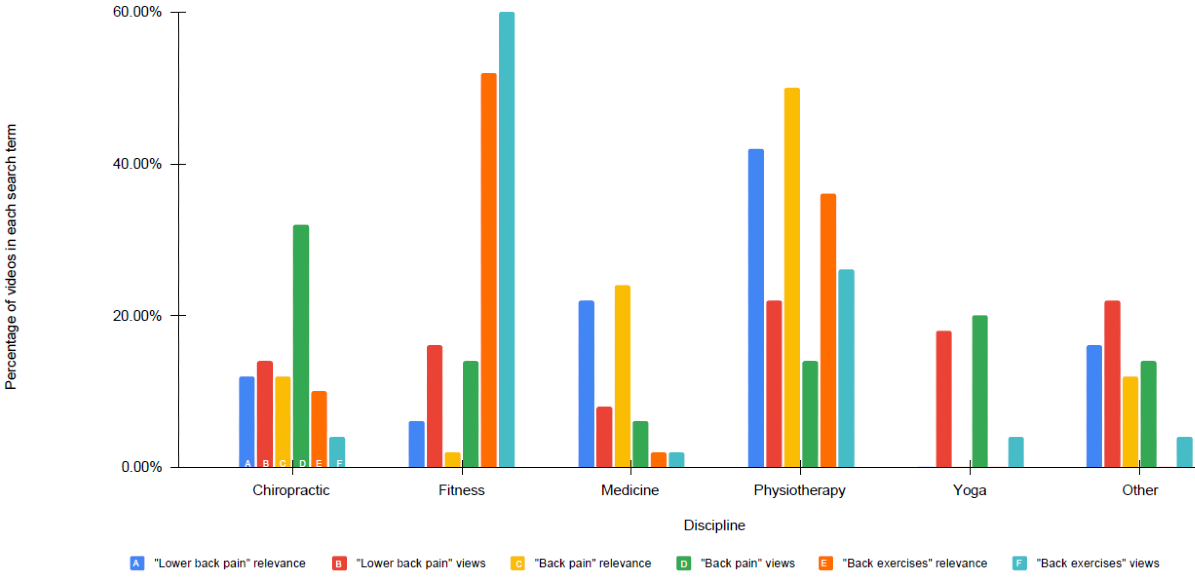

Supplement: Multimedia Appendix 3 [file formative_v4i11e23366_app3.png]

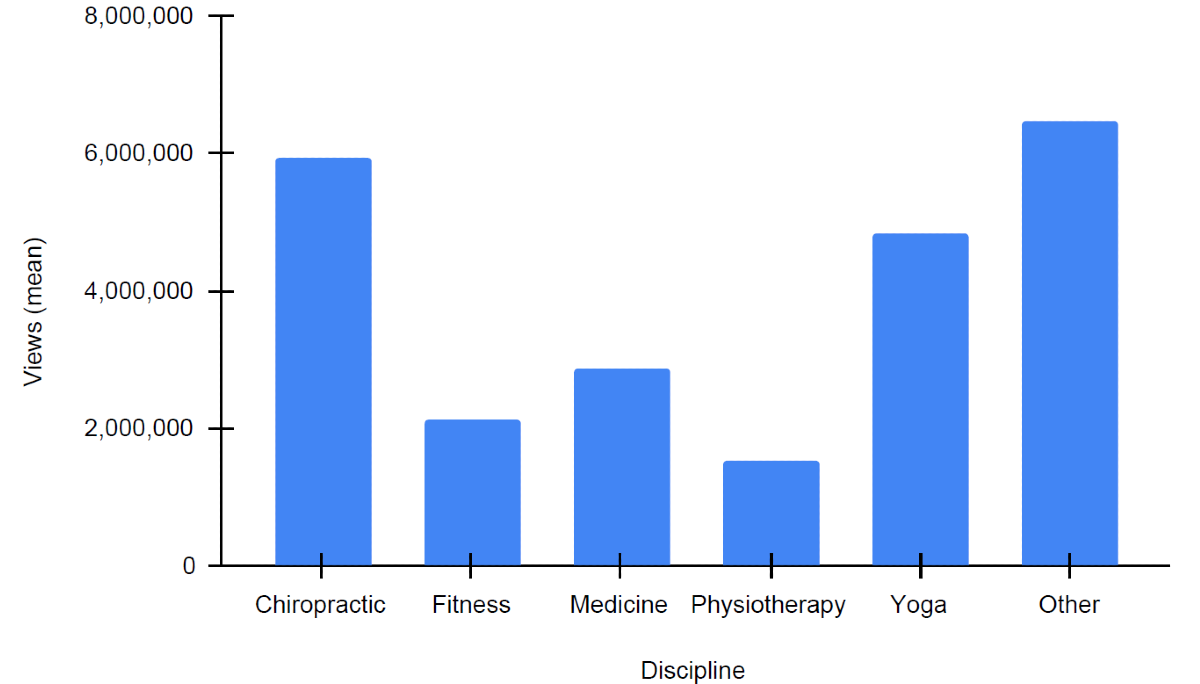

Supplement: Multimedia Appendix 4 [file formative_v4i11e23366_app4.png]

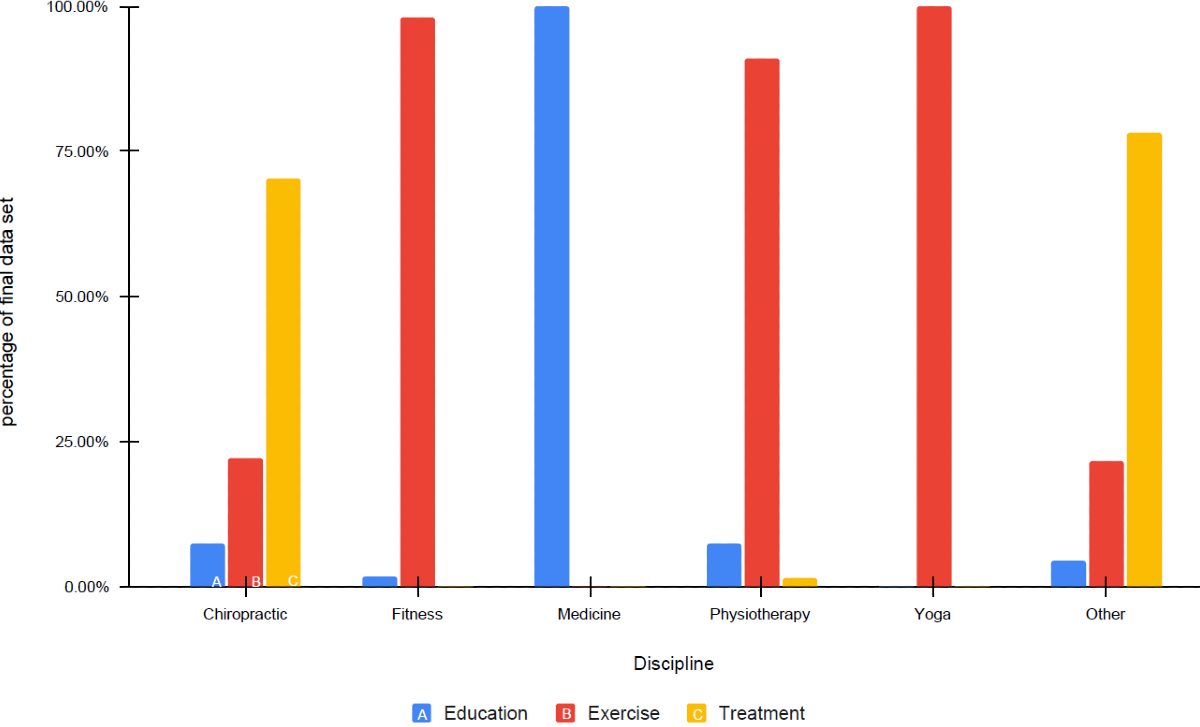

Supplement: Multimedia Appendix 5 [file formative_v4i11e23366_app5.png]

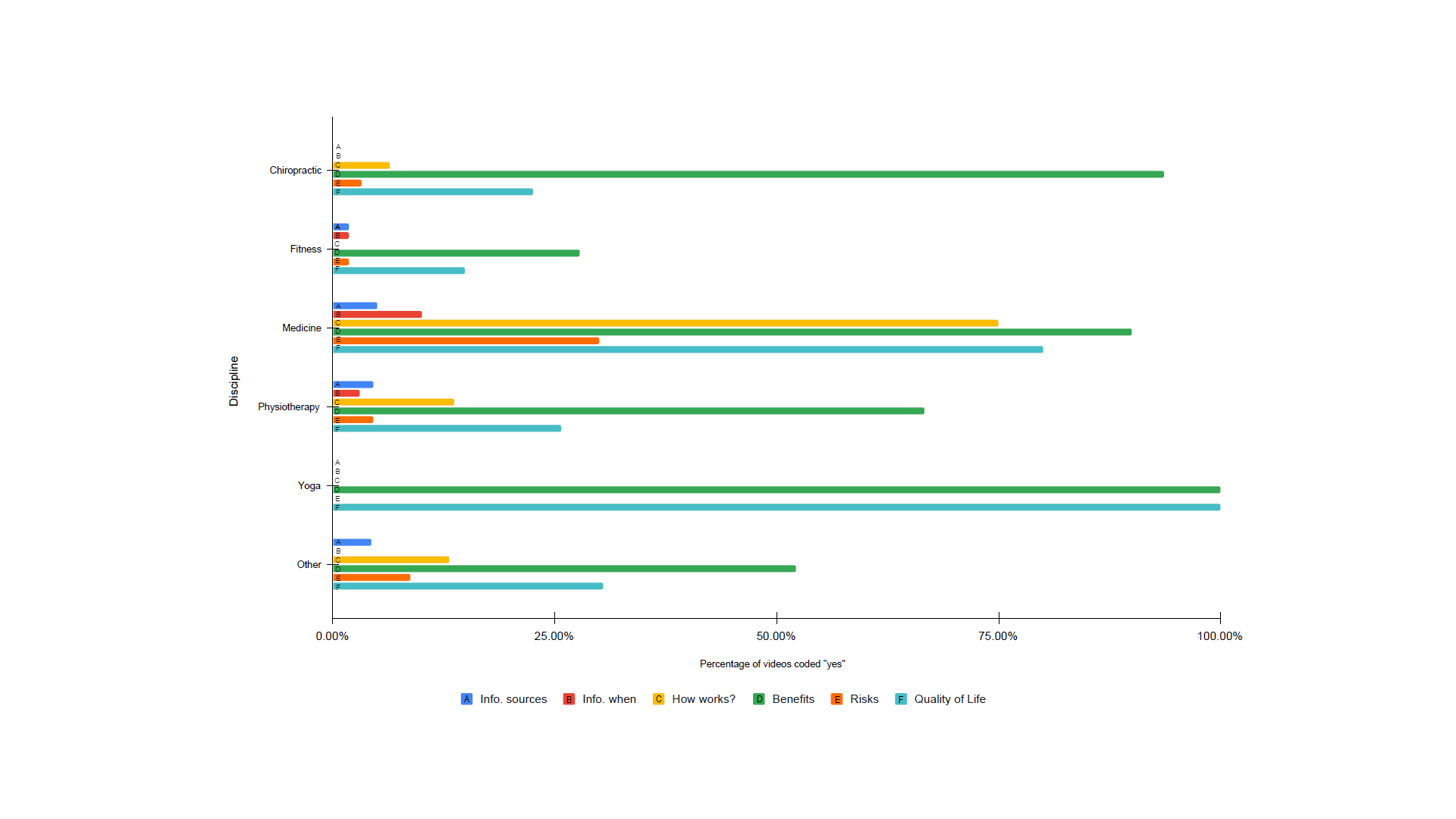

Supplement: Multimedia Appendix 10 [file formative_v4i11e23366_app10.png]

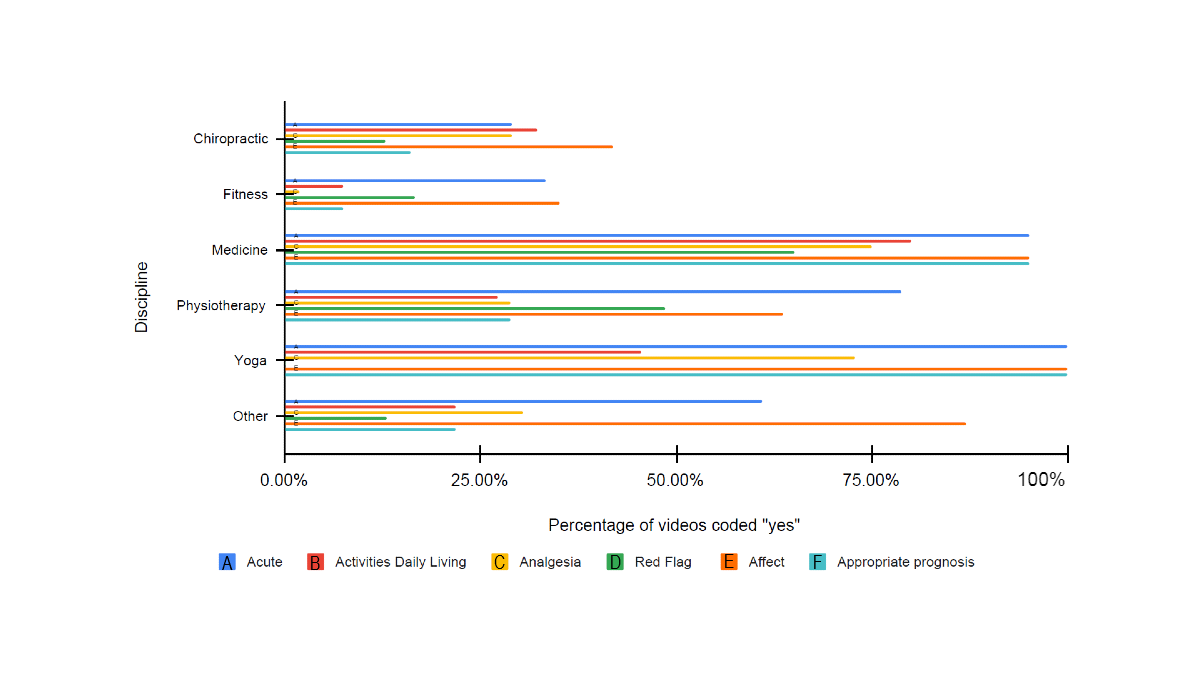

Supplement: Multimedia Appendix 11 [file formative_v4i11e23366_app11.png]
