# Supplementary material for: Plausibility of Using a Checklist With YouTube to Facilitate the Discovery of Acute Low Back Pain Self-Management Content: Exploratory Study
Source: JMIR Form Res. 2020 Nov 20;4(11):e23366. doi: 10.2196/23366 (PMC7718094; doi:10.2196/23366)
Supplement: Multimedia Appendix 6 [file formative_v4i11e23366_app6.pdf]

Appendix 2: Modified Brief Discern codebook

Brief Discern question	Brief Discern codebook
1. Is it clear what sources of information were used to compile the publication (other than the author or producer)?	**YES:** At least one specific peer reviewed reference mentioned in the video.
	**NO:** No specific peer reviewed reference mentioned in the video.
5. Is it clear when the information used or reported in the publication was produced?	**YES:** Specific date of a peer reviewed reference mentioned in the video (not publication date of video itself).
	**NO:** No specific publication date of a peer reviewed reference mentioned in the video.
9. Does it describe how each treatment works?	**YES:** Description of the treatment should be based on current mainstream biologically plausible theory. Eg biopsychosocial pain theory.
	**NO:** Argument for video content founded on arguments incorporating terms such as tension, release, stretch, swelling, adjustment of discs, leg length, and spinal rotation.<br>OR<br>**No** description of how treatment works
10. Does the publication describe the benefits of each treatment?	**YES**: At least one benefit for low back pain or disability resulting from the activity described in the video - eg pain, disability, emotional state.<br>No evidence is required.
	**NO:** No benefits for low back pain described
11. Does it describe the risks of each treatment?	**YES;** At least one medical risk of the intervention described in the video content (eg stroke, paralysis etc) This excludes minor temporary local soreness.
	**NO:** no medical risks of the intervention described in the video.
13. Does it describe how the treatment choices affect overall quality of life?	**YES;** There is at least one broad statement describing the overall effect of treatment - ie not just reduced back pain or disability.
	**NO:** No mention of influence of the intervention on the overall quality of life.
