# Supplementary material for: Plausibility of Using a Checklist With YouTube to Facilitate the Discovery of Acute Low Back Pain Self-Management Content: Exploratory Study
Source: JMIR Form Res. 2020 Nov 20;4(11):e23366. doi: 10.2196/23366 (PMC7718094; doi:10.2196/23366)
Supplement: Multimedia Appendix 7 [file formative_v4i11e23366_app7.pdf]

Appendix 3: ALBP Checklist

**ALBP Checklist**	**ALBP codebook - definitions**
1.Acute (Suitable for acute patients)	YES: The exercises and advice are of appropriate intensity and scope for early onset low back pain in a middle aged individual of average fitness and flexibility level. Yoga, pilates, and walking are all equally effective for example.
	NO: The exercises and advice are not suitable as described above
2. ADL (Activities of daily living)<br><br>Lancet:<br>"Advice to remain active"	YES: At least ONE mention of:<br>Activities of daily living (ADL) during video<br>This includes walking, modifying environment, sport and recreation, modifying ADL, maintaining ADL.
	NO: No mention of ADL as described above
3. Analgesia	**YES:** At least one mention of:<br>1) Over the counter pharmacological analgesia<br>OR<br>2) non-pharmacological self-management described in guidelines (for example NSAIDS, heat, ice, pacing, effect of exercises on pain)
	**NO:** No mentions of pharmacological or non-pharmacological analgesia
4. Reassuring affect	**YES:** Is the video emotionally reassuring overall?<br>This includes: education content, editing, music, encouraging fear or catastrophizing, and/or loud, urgent, or frantic narration.
	**NO:** Not reassuring overall as per description of reassuring affect as described above.
5. Appropriate Prognosis	**YES:** Author describes:<br>1) Plausible prognosis consistent with Lancet non-specific acute low back pain guidelines.<br>Not overpromising.
	**NO:**<br>1) Overpromising.Terms such as "fix", "cure" "instant" indicate unrealistic prognosis. Also discouraging imaging..<br>OR<br>2) No prognosis mentioned.
6. Red flags	**YES**: At least one mention of referral to mainstream medical provider for formal advice in video content.<br>OR<br>Mention of alternative medical diagnosis or symptoms including: cauda equina, renal problems, abdominal aortic aneurysm, cancer, infection
	**NO:** No mention of referral to mainstream medical provider for formal advice in video content<br>OR<br>No mention of medical diagnosis for low back pain
