# Supplementary material for: Plausibility of Using a Checklist With YouTube to Facilitate the Discovery of Acute Low Back Pain Self-Management Content: Exploratory Study
Source: JMIR Form Res. 2020 Nov 20;4(11):e23366. doi: 10.2196/23366 (PMC7718094; doi:10.2196/23366)
Supplement: Multimedia Appendix 8 [file formative_v4i11e23366_app8.pdf]

Appendix 6 Final data set

**Chiropractic videos**				
Channel name	YouTube URL	Discipline	Length Seconds	Views
Chiro Core	T6DQrzMJZE4	Chiropractic	721	2509409
Dr. Rahim Gonstead Chiropractor	lTBIjCA_F7E	Chiropractic	1923	12887206
Advanced Chiropractic Relief	MgobNg4DGQk	Chiropractic	685	89413
Chiro Core	1hEDlRpm1jg	Chiropractic	323	8289676
Chiro Core	EhzLv5tlxGY	Chiropractic	379	22234444
Discover Chiropractic	gz4X01_myVE	Chiropractic	341	3516166
Dr Joseph Cipriano DC	K43e7seYTZk	Chiropractic	792	2289167
Dr Joseph Cipriano DC	KKjSS9Ig7_A	Chiropractic	980	3605381
Dr Joseph Cipriano DC	QzLuTgx5h5I	Chiropractic	678	3175118
Back Intelligence	vuGnzLxRvZM	Chiropractic	309	168763
DrJason	_FuPD0HXxXA	Chiropractic	230	58136352
Dr Joseph Cipriano DC	iTlNA_4nDa0	Chiropractic	728	2359384
Dr Joseph Cipriano DC	Jg-0cIXZBoM	Chiropractic	902	17276289
Advanced Chiropractic Relief LLC	dXh_LueAddo	Chiropractic	498	258767
Dr. Kamilla Holst	q75OrnukwbA	Chiropractic	284	4734194
Dr. Rahim Gonstead Chiropractor	FrfZ2Yo9QrE	Chiropractic	559	7033459
Dr. Rahim Gonstead Chiropractor	jWPMVoH1wFU	Chiropractic	1346	4874844
ACTIV CHIROPRACTIC	CdCClhtKH2Q	Chiropractic	493	1643391
ACTIV CHIROPRACTIC	PZfyQIoxJIQ	Chiropractic	336	7550
ACTIV CHIROPRACTIC	GjHMoj3HT8I	Chiropractic	202	410349
ACTIV CHIROPRACTIC	8p9TBuIMmIo	Chiropractic	311	200831
Advanced Chiropractic Relief LLC	lho-8MCvvnQ	Chiropractic	660	1569664
Advanced Chiropractic Relief LLC	dA0zGUK4N1U	Chiropractic	714	60736
Advanced Chiropractic Relief LLC	jl1NkJSIKdo	Chiropractic	809	5333096
Back Intelligence	2a2osZ_1FVs	Chiropractic	245	1436894
ACTIV CHIROPRACTIC	6RX3OiwFqP8	Chiropractic	179	247295
ACTIV CHIROPRACTIC	t_uR01Dx9Mk	Chiropractic	190	1456610
Back Intelligence	bGAz_p7nUbY	Chiropractic	456	4528
			581.18	5,921,749.14
			Mean length	Mean views
**Fitness videos**				
Channel name	YOUTUBE URL	Discipline	Length Seconds	Views
Stanford	mdJStrJ5t9g	Fitness	1428	24242
Richard Ji	_qjBvQ9F4kw	Fitness	226	1372035
Hanna Öberg	6SYlMgVgm50	Fitness	622	325588
Jeremy Ethier	8LJ3Q3Fsrzs	Fitness	568	4909718
Gravity Transformation	apWQucmQU5w	Fitness	661	3534856
SixPackAbs.com	b5fmea4D_3Q	Fitness	345	1382475

Simeon Panda	E0JNbBccexE	Fitness	432	404138
Mike Thurston	Eh93DTXfahk	Fitness	689	2810850
Sparta Strength	F7-cBttLavU	Fitness	331	3128417
Bodybuilding.com	Fe7pzbZQ0Gs	Fitness	597	1783185
BroScienceLife	G5GIJmCwchk	Fitness	379	3772361
Anabolic Aliens	JQeOhQoi3GY	Fitness	375	2946508
Jeremy Ethier	kBWAon7ItDw	Fitness	571	1178794
itzDeaMan	KSZogPDS9c0	Fitness	207	2123310
Bodybuilding.com	lA7dbOmxs5I	Fitness	507	7017928
Bodybuilding.com	mcCg_ycMhlA	Fitness	262	5181805
Simeon Panda	NoRkALo4AMQ	Fitness	466	3387144
CHRIS HERIA	qqEw8XH-feI	Fitness	616	388118
Love Sweat Fitness	qqHJLTi2yX4	Fitness	403	1576818
GYM BODY MOTIVATION	s8taXR3mYa8	Fitness	274	1475965
musclemonsters	StbBFiyNimA	Fitness	569	263247
UlissesWorld	T60YhWfv9no	Fitness	622	174640
Krissy Cela	twjhKJtHwfg	Fitness	530	975218
V Shred	upYjtQEbjAI	Fitness	838	214044
WORKOUT	vNzxQN-1kDI	Fitness	516	827729
ePainAssist	WFktLpyDprM	Fitness	219	2194951
AlphaShred TV	xrsDAn7RJ8U	Fitness	340	549232
Health And Fitness	Xvddna84BXo	Fitness	774	2252317
Christina Carlyle	y70jB93squQ	Fitness	512	2773682
PROMiXX	YpNu9vNePSo	Fitness	184	553445
Mike O'Hearn	yXVouZ84Mvg	Fitness	216	2233315
Beauty recipes	z8LOO9_OdZE	Fitness	216	2233372
Gabriella Whited	EwfY0gz9a1I	Fitness	399	6812080
Roberta's Gym	doy7c0eumus	Fitness	677	4375129
BRIGHT SIDE	DXBAq5taLs0	Fitness	1058	2604871
Redefining Strength	E5w3nZpvdI0	Fitness	603	6646
Posture Makeover	0OkB0xO_9kU	Fitness	807	3857338
Sharp HealthCare	5nZ8n1vUqVU	Fitness	60	916252
FitnessBlender	YMwi_eDYG9U	Fitness	1517	1219229
FitnessBlender	CbZzeO4P9YA	Fitness	537	2401939
Memorial Healthcare System	q-FlsD3fubA	Fitness	129	430560
Kai Simon	dl474z1bhnk	Fitness	299	1934607
Howcast	MnWWDAsEfXk	Fitness	172	1859565
Calisthenicmovement	qAubcPOQw3s	Fitness	305	2656641
FitnessBlender	ahPse5W9Eyo	Fitness	1074	1934513
FitnessBlender	p1GERX3lf3Y	Fitness	481	2726271
Jeremy Ethier	2tnATDflg4o	Fitness	596	1430078
Living Better	pnn-uOtO6aI	Fitness	251	285513

FitnessFAQs	ZlY9Y_nQ2V8	Fitness	317	1686373
HASfit	b4ZnvU3QkRM	Fitness	1650	1314688
BRIGHT SIDE	g-sen-A0rtg	Fitness	431	2563955
Matty Fusaro	HoNW3TnAT3Y	Fitness	369	2974439
HASfit	p6CMso14NWk	Fitness	1247	4866508
BlackBeltSecrets	7R3GDSHS_y8	Fitness	281	3917171
HASfit	_lT5Cd4cDTc	Fitness	1990	24125
			559	2123053.418
			Mean length	Mean views
**Medical videos**				
Channel name	YOUTUBE URL	Discipline	Length Seconds	Views
BMI Healthcare	4HwtjtR3b_M	Medical	205	79541
nabil ebraheim	zfWKcTixM6E	Medical	363	2190472
Nucleus Medical Media	nV4ILsaVSXc	Medical	250	2009304
nabil ebraheim	AWR1M90NG5s	Medical	363	2889806
UCLA Health	vRS0je8eopc	Medical	53	220824
DoctorOz	-MIdGP4i1bA	Medical	313	2251415
UW Medicine	n_E76I25_Vg	Medical	140	476409
Dartmouth-Hitchcock	nizukO1o2AU	Medical	137	283221
UC San Diego Health	20zybMbnVoU	Medical	321	4863685
Quebec Pain Research Network	DkRnZNAnWfE	Medical	365	2001520
Coordinated Health	Lk8eIJCLpQg	Medical	493	4155470
UPMC	UANvh8QynLI	Medical	176	1005938
Carilion Clinic	7LPUUqJxDyE	Medical	265	4806392
OHSU	sy41zRUYp48	Medical	156	14261
Coordinated Health	WGY6GDJUBPY	Medical	493	792615
Duke Health	1HXrLxAVfOg	Medical	349	4286439
Carilion Clinic	2aNcuZbm7N0	Medical	265	4806392
Top Doctors UK	7Vyiyynzv48	Medical	319	587114
DocMikeEvans	BOjTegn9RuY	Medical	666	16887686
			299.58	2,874,131.79
			Mean length	Mean views
**Physiotherapy videos**				
Channel name	YOUTUBE URL	Discipline	Length Seconds	Views
ATHLEAN-X™	a9_JPgwW9nw	Physiotherapy	700	2305800
ATHLEAN-X™	eE7dzM0iexc	Physiotherapy	636	3675787
ATHLEAN-X™	gIhCuqtC0r0	Physiotherapy	508	3697398
ATHLEAN-X™	mjnseqLiVXM	Physiotherapy	266	3607231
ATHLEAN-X™	NhlVYy1wkKQ	Physiotherapy	444	1960804
ATHLEAN-X™	nK3BNozPEx4	Physiotherapy	418	2806115
ATHLEAN-X™	oLwTC-lAJws	Physiotherapy	841	7114831
ATHLEAN-X™	OXvQe9payHw	Physiotherapy	670	6271510

ATHLEAN-X™	TXh1oLXb6zk	Physiotherapy	398	1718744
ATHLEAN-X™	ydT74SAts7M	Physiotherapy	346	1716731
Bob & Brad	fBpYx1XsMqg	Physiotherapy	719	3678294
Bob & Brad	jogQL7TxXTA	Physiotherapy	730	1306446
Madden PT	sDenSUIGuek	Physiotherapy	176	2208299
KT Tape	tPWau00bAYE	Physiotherapy	148	198266
AskDoctorJo	lCxDDAWIi2Q	Physiotherapy	356	260119
Michelle Kenway	QB93HgZJiio	Physiotherapy	255	129635
Michelle Kenway	TGI5TFnY8Ck	Physiotherapy	283	960316
Michelle Kenway	u_YaHaTg5aE	Physiotherapy	391	1679358
AskDoctorJo	UYMmtEFhuxA	Physiotherapy	719	95026
AskDoctorJo	wgPf9IJiW5s	Physiotherapy	281	126901
AskDoctorJo	WRRFUP_pklI	Physiotherapy	369	10942
ATHLEAN-X™	DWmGArQBtFI	Physiotherapy	563	16924450
ATHLEAN-X™	IZcOAQoE81A	Physiotherapy	403	12352
AskDoctorJo	cRfadxPlVNs	Physiotherapy	456	126867
Bob & Brad	6HOvGr6Tqa8	Physiotherapy	653	2099028
Madden PT	htgyPKNHUls	Physiotherapy	260	4216315
AskDoctorJo	hzv6NvPW33Q	Physiotherapy	633	76540
ChoosePT	iVyVnbESt1s	Physiotherapy	165	64614
AskDoctorJo	Jn8nYOM6dug	Physiotherapy	269	9961
Physio Fitness	hjpdXzVlAiU	Physiotherapy	665	38172
AskDoctorJo	4aR-v_5f-T4	Physiotherapy	429	113550
AskDoctorJo	BoMiM_mnr1Y	Physiotherapy	315	2008087
AskDoctorJo	bTn89EBKJdM	Physiotherapy	179	71880
Active Rehabilitation Physiotherapy	i_ohtG0DKrw	Physiotherapy	237	124075
Michelle Kenway	tDbvVITtpFU	Physiotherapy	283	408915
AskDoctorJo	wrrOCg6V-qU	Physiotherapy	544	346507
AskDoctorJo	Xm8iSSdU3I0	Physiotherapy	447	1468091
AskDoctorJo	ykeLv6yEvNs	Physiotherapy	315	2008087
Michelle Kenway	TVosLzpgT64	Physiotherapy	239	561052
AskDoctorJo	bEDH_uTcdf4	Physiotherapy	499	396597
AskDoctorJo	qFaarJsrJFQ	Physiotherapy	490	11744
Sharp HealthCare	HqwXzi8xpXE	Physiotherapy	187	752128
Ask Dr Jo	n6Etl3x9AnU	Physiotherapy	544	338246
AskDoctorJo	750nkDg9XPI	Physiotherapy	450	136362
AskDoctorJo	8oPHrX_oALk	Physiotherapy	246	2488737
Bob & Brad	clfpWjqVP6U	Physiotherapy	465	1836199
Physiotutors	QVBxB59Y4Y4	Physiotherapy	276	18127
Bob & Brad	8MyjVqDxfSc	Physiotherapy	340	4349871
AskDoctorJo	S3xXurLpfDk	Physiotherapy	1162	498733
ATHLEAN-X™	bOJu7xi3l3Q	Physiotherapy	582	2756896

CHI Health	0_4mojKLL3U	Physiotherapy	81	11558
Bob & Brad	1RBO6FZBK14	Physiotherapy	721	13903
AskDoctorJo	9S2SPTfh8NE	Physiotherapy	471	16718
ATHLEAN-X™	9SKuFe2SERs	Physiotherapy	531	5258843
AskDoctorJo	2VuLBYrgG94	Physiotherapy	328	1315777
Duke Health	imM1CdW41A8	Physiotherapy	173	598971
Michelle Kenway	9j0wTLcElUg	Physiotherapy	283	405483
Michelle Kenway	fVEd8M_ZSdQ	Physiotherapy	191	23349
Lee Health	0mXJ7Up7kcs	Physiotherapy	104	300024
Tone and Tighten	-Qxmc4E7IwA	Physiotherapy	735	2280032
Children's Hospital Colorado	bug-Eg27s2I	Physiotherapy	146	203655
TEDx Talks	ErmOTERAnQo	Physiotherapy	874	34578
SingHealth	zLCae7TQx_4	Physiotherapy	253	327435
The McKenzie Institute, USA	gsUE0CEz6g0	Physiotherapy	2608	127848
Ohio State Wexner Medical Center	9dNfZYfbVq4	Physiotherapy	105	9625
AskDoctorJo	2yCzNbWQTjo	Physiotherapy	610	55656
			457.03	1,526,881.68
			Mean length	Mean views
**Yoga videos**				
Channel name	YOUTUBE URL	Discipline	Length Seconds	Views
Yoga With Adriene	d6zJkHcjbWc	Yoga	723	1800366
PsycheTruth	9f1Y4OV3QlA	Yoga	843	5,234,392
PsycheTruth	Ub8afedu5GQ	Yoga	1427	3242287
Natasha Noel	ImHDTDhxSpE	Yoga	782	8847370
PsycheTruth	HpFCxqYwwJc	Yoga	1149	3131943
Yoga With Adriene	Ho9em79_0qg	Yoga	1400	4224230
Yoga With Adriene	phuS5VLQy8c	Yoga	1874	6658817
Yoga With Adriene	XeXz8fIZDCE	Yoga	947	4863736
PsycheTruth	CO3racIlTcg	Yoga	1845	7680423
PsycheTruth	Q00u-60XM9Y	Yoga	2441	4319510
YOGATX	pyFNz8zJSdw	Yoga	1971	3039986
			1,400.18	4,822,096.36
			Mean length	Mean views
**Other videos**				
Channel name	YOUTUBE URL	Discipline	Length Seconds	Views
Lex Fitness	edZaTzJ1fI0	Other	854	1933962
Traditional Massage Official	kjMKXY0pMy0	Other	654	32688519
John Gibbons	O86oNfz8Peg	Other	126	347710
John Gibbons	wAxw3P61_1w	Other	153	572751
John Gibbons	y8poqChYAes	Other	172	2763851
John Gibbons	ZLXYVHpSYgM	Other	157	415767
MassageASMR	tsjIGmLtz_I	Other	1444	7497487

Thai Massage Services	_aFYOB8sj4Q	Other	683	30067303
MassageASMR	tsjIGmLtz_I	Other	1444	7484521
MassageASMR	zNCVrO6nlR8	Other	1836	4386842
ePainAssist	UogxbusNOVo	Other	146	2827782
Melbourne Muscular Therapies	blZsRh6_guo	Other	350	10420920
DJO ANZ	WwqAjrYGfq0	Other	237	9486
John Gibbons	BXHr7l4l4_Y	Other	191	9394
Vitality Massage	bxOBt3XKsoA	Other	840	1445999
BRIGHT SIDE	QDFFKOtuHh0	Other	943	2661822
BRIGHT SIDE	x_WJ5cotyfE	Other	785	7005327
Carrington College	zNZ0JLHGxAY	Other	427	1435990
Intermountain Healthcare	V7tWWdbOYtQ	Other	277	790617
Thai Massage Services	UqTgp_3h8As	Other	2112	22443610
Thai Massage Services	nyszoKVMha0	Other	1558	6502940
AbrahamThePharmacist	1Yq_yehKReo	Other	272	109215
Info Tech	WpjJECNiBxs	Other	417	4890830
			699.04	6,465,767.17
			Mean length	Mean views
