# Supplementary material for: Plausibility of Using a Checklist With YouTube to Facilitate the Discovery of Acute Low Back Pain Self-Management Content: Exploratory Study
Source: JMIR Form Res. 2020 Nov 20;4(11):e23366. doi: 10.2196/23366 (PMC7718094; doi:10.2196/23366)
Supplement: Multimedia Appendix 9 [file formative_v4i11e23366_app9.pdf]

# Appendix 7 Most viewed video in final data set.

| Most viewed video in data set | |
|---|---|
| Title: | DR. JASON-YEARS Of Back PAIN Helped With CHIROPRACTIC Care |
| URL: | https://www.YouTube.com/watch?v=_FuPD0HXxXA |
| Discipline: | Chiropractic |
| Length (seconds) | 229 |
| Views (as at March 2020) | 58136352 |
